# Dry eye disease in astronauts: a narrative review

**DOI:** 10.3389/fphys.2023.1281327

**Published:** 2023-10-19

**Authors:** Timon Ax, Bergita Ganse, Fabian N. Fries, Nóra Szentmáry, Cintia S. de Paiva, Francesc March de Ribot, Slade O. Jensen, Berthold Seitz, Thomas J. Millar

**Affiliations:** ^1^ Department of Ophthalmology, Saarland University Medical Center, Homburg/Saar, Germany; ^2^ School of Medicine, Western Sydney University, Sydney, NSW, Australia; ^3^ Werner Siemens-Endowed Chair for Innovative Implant Development (Fracture Healing), Departments and Institutes of Surgery, Saarland University, Homburg/Saar, Germany; ^4^ Department of Trauma, Hand and Reconstructive Surgery, Departments and Institutes of Surgery, Saarland University, Homburg/Saar, Germany; ^5^ Dr. Rolf M. Schwiete Center for Limbal Stem Cell and Aniridia Research, Saarland University, Homburg/Saar, Germany; ^6^ Ocular Surface Center, Department of Ophthalmology, Baylor College of Medicine, Cullen Eye Institute, Houston, TX, United States; ^7^ Department of Ophthalmology, Otago University, Dunedin, New Zealand; ^8^ Antimicrobial Resistance and Mobile Elements Group, Ingham Institute of Applied Medical Research, Sydney, NSW, Australia; ^9^ Beyond 700 Pty Ltd, Sydney, NSW, Australia

**Keywords:** dry eye, tear film, international space station, blink, eyelid, lacrimal duct, microgravity, spaceflight

## Abstract

Long-duration spaceflight can have adverse effects on human health. One of the most common ocular conditions experienced by astronauts is dry eye disease (DED). Symptoms of DED include feelings of eye irritation, eye strain, foreign body sensation and blurred vision. Over 30% of International Space Station expedition crew members reported irritation and foreign body sensation. We reviewed the current literature on the prevalence and mechanisms of DED in astronauts and its potential implications for long-duration spaceflight, including the influence of environmental factors, such as microgravity and fluid shift on tear film physiology in space. DED has negative effects on astronaut performance, which is why there is a need for further research into the pathophysiology and countermeasures. As an in-flight countermeasure, neurostimulation seems to be among the most promising options.

## 1 Introduction

Upon return to Earth, over 30% of International Space Station (ISS) expedition crew members and approximately 15% of shuttle crew members reported experiencing signs or symptoms of dry eye disease (DED) while in space (Gibson and Duncan, 2009; Reschke et al., 2016). DED is an umbrella term encompassing a variety of diseases affecting the ocular surface with tear film dysfunction and inflammation as their shared feature (Aggarwal and Galor, 2018). Common symptoms include eye strain, burning or stinging, grittiness, itchiness, redness, sensitivity to light, and blurry and reduced vision, all of which are frequently reported by astronauts (Szczotka-Flynn et al., 2019; Meer et al., 2023). In the US, a recent meta-analysis estimated a DED prevalence of 8.1% for the entire population (McCann et al., 2022). Given the medical testing and excellent health of spaceflight participants prior to flights, the high incidence of DED might be related to the microgravity exposure. Sleep and mood disorders associated with DED have been reported on Earth (Ayaki et al., 2016; Kitazawa et al., 2018; Zhou et al., 2022), and are also both known to affect crew performance (Wu et al., 2018; Stuster, 2021). Thus, on long-duration flights, DED will need to be addressed because visual acuity and comfort are critical for performing space-related tasks. However, the pathophysiology of DED in microgravity and possible countermeasures are unknown. Despite its high incidence and operational relevance, DED was not mentioned in the latest consensus paper concerning the directions to identify risks and mitigation strategies for ocular changes in human spaceflight (Seidler et al., 2022). For these reasons, the aim of the present review paper was to summarize the current evidence for the clinical relevance, pathophysiology, and possible countermeasures of DED in microgravity.

## 2 Physiology of the tear film

The tear film is a thin fluid film covering the cornea and bulbar conjunctiva. The three main tear film components are mucins, water, and lipids (Yokoi et al., 2014). The mucins are embedded in the apical membrane of ocular surface cells and are also secreted into the tear film by specialized conjunctival goblet cells (Spurr-Michaud et al., 2007; Hodges and Dartt, 2013). The aqueous portion of the tear film comprising a mixture of proteins, water, and ions is secreted by the lacrimal glands (Green-Church et al., 2008) and the lipids are secreted by glands (Meibomian glands) in the eyelids (Bron et al., 2004). Besides maintaining sharp vision, the tear film keeps the eye healthy by providing microbial protection and nutrients (McDermott, 2013).

## 3 Tear film dynamics and effects of microgravity

### 3.1 Tear film structure

In the scientific community, current consensus is that the tear film should be regarded as a whole, rather than three separate layers (Georgiev et al., 2019). Together, the three layers combined enable the final tear film to be only ∼ 3 µm thick, and to cover a relatively vast area (∼2 cm^2^; or ∼2 × 10^8^µm^2^) without collapsing (Tsubota and Nakamori, 1995). This means that molecular rather than gravitational forces dominate its spread and stability (Jones et al., 2005). However, gravity is likely to have a direct impact on the tear menisci, i.e., the thin bands of tear fluid between the eyeball and lid margins. Gravitational effects cause a steepened curvature and lower tear volume in the upper meniscus and *vice versa* in the lower meniscus (Su et al., 2015; Maki et al., 2020). It is, however, unclear whether changes in tear film components in space are responsible for the DED complaints reported by astronauts. Neither tear film sampling of astronauts, nor analyses of tear film spread have been performed to date.

### 3.2 Blinking

Blinking is a cycle of movement that helps to distribute and remove the tear film. The opening part of the blink cycle is responsible for spreading the aqueous and meibum (lipid) to form an even tear film over the eye (Harrison et al., 2008). The closing phase of a blink (∼100 ms) is faster than the opening phase (∼200 ms) (Kwon et al., 2013; Mak et al., 2016; Rodriguez et al., 2018; McMonnies, 2021). Tear film spreading is completed in ∼1 s after cessation of the upstroke of the blink (Ring et al., 2012). During the open-eye part of the blink cycle, the tear film evidently forms a gel and becomes isolated from the reservoir of tears in the upper and lower tear menisci (Miller et al., 2002; Yokoi et al., 2014; Georgiev et al., 2019). Lubricity between the eyelid and ocular surface in part is maintained by specialized stratified epithelium that covers the lid margin at the point of closest contact (lid wiper). Changes to this region of the eyelid have been associated with signs of poor tear film performance (Korb et al., 2005).

The fast-closing part of the blink cycle is needed to fluidize the tear film because it shows non-Newtonian viscosity i.e., the higher the shear force the less viscous the tears (Pult et al., 2015b; Arshinoff et al., 2021; Recchioni et al., 2022). Blinking also cleans the ocular surface by removing tears via the tear menisci into the puncta, lacrimal canaliculi and lacrimal sac (McDonald and Brubaker, 1971). Dust and microbe removal from the eye surface is likely to be even more important in microgravity where large dust particles and dense objects such as metal filings do not settle (Haines et al., 2019; Jahn et al., 2021). In fact, astronauts commonly report corneal foreign bodies (Meer et al., 2023).

Blink speed, frequency and fullness contribute to normal tear film formation. Studying the nature of blinking is difficult because of differences in defining a complete/full blink (Collins et al., 2006; Pult et al., 2013; Wang et al., 2018) and blink frequency varies with alertness, conversation, the nature of visual task being undertaken, mood, sex, and ethnicity (Yolton et al., 1994; Tsubota et al., 2002; Himebaugh et al., 2009; Wang and Craig, 2019). Natural blinking changes upon conscious command, and the characteristics of purposeful blinking are very individual (McMonnies, 2020). Eyelid contact in full blinks compresses the Meibomian glands, increases secretion and spreads oils onto the eyelid margins (Korb et al., 1994). Nevertheless, as little as 10% of blinks need to have the lids fully touching along their length (full in amplitude), to maintain a stable tear film (Shew et al., 2022). Mathematical and analog models of blinking have not been able to predict all the clinical observations (Braun and Fitt, 2003; Pult et al., 2015a). While it is known that being in microgravity significantly affects gaze behavior via the vestibular system, blink behavior has not yet been studied (Clément, 2011; Reschke et al., 2016). Given the above, we hypothesize that if blinking remains normal the tear film should form properly in microgravity.

### 3.3 The effect of cephalad fluid shift (CFS) on the tear film

In microgravity, fluid redistributes from the lower half of the body to the head causing tissue swelling and increases in intraocular and cranial pressure (Nelson et al., 2014). The effects of cephalad fluid shift (CFS) have been well studied in relation to retinal thickness, axial length, anterior chamber depth, and refraction, and the changes to eye health have been labeled as spaceflight-associated neuro-ocular syndrome (SANS) (Lee et al., 2020; Macias et al., 2020). CFS also causes swelling of the eyelids and an upward shift in eyebrow position (Schneider et al., 2016; Karlin et al., 2021). Thereby, the nasolacrimal drainage apparatus is affected. Swelling of the eyelids may change the pressure that they exert onto the ocular surface (lid wiper), and the size and shape of the tear menisci; both could influence the spread of the tear film (Yamamoto et al., 2016; van Setten, 2020). Swelling will affect tear drainage because normally in the open eye, the upper and lower canaliculi are relaxed and fill with aqueous. The common canaliculus is constricted and then, during a blink, the aqueous flows into the common canaliculus because the upper and lower canaliculi constrict and the common canaliculus relaxes (Kakizaki et al., 2013). From there, the tears flow into the lacrimal sac which is surrounded by a cavernous body. It is rich in a vascular plexus that is important for regulating tear-outflow by dilating and constricting (Paulsen et al., 2000; Ayub et al., 2003). CFS is expected to change the vascular plexus of the cavernous body resulting in functional nasolacrimal duct obstruction and tear retention. Tear drainage from the lacrimal sac to the inferior meatus of the nose is also affected by gravity (Chavis et al., 1978; Sahlin and Chen, 1997). Insufficient drainage means the tear film is not replaced leading to the accumulation of toxins and inflammatory agents (Pflugfelder et al., 2002; de Paiva and Pflugfelder, 2004). Partial gravity (e.g., Moon or Mars gravity) is expected to induce similar changes but to a lesser degree.

## 4 Evaluation of tear film problems in space

Diagnosing tear film disorders on Earth has proven to be cumbersome because the tear film is complex, invisible, and small in volume. Sampling and bright light causes reflex tearing, and there is discordance between signs and symptoms of DED (Behrens et al., 2006; Sullivan et al., 2014; Bartlett et al., 2015; Wolffsohn et al., 2017), e.g., symptoms have been associated with non-ocular pain, depression, and post-traumatic stress disorder (Galor et al., 2015). Moreover, different clinics vary in their cut-off values to distinguish a normal tear film from an unhealthy one. Consequently, diagnosis is often uncertain and inconsistent. To minimize this problem, the TFOS DEWS II (Dry Eye Workshop) expert panel recommended an initial questionnaire, followed by tests for any one of three possible markers of a loss of tear film homeostasis (tear film stability, hyperosmolarity or corneal staining). Some examples are non-invasive breakup time (NIBUT), osmolarity, and ocular surface staining with fluorescein and lissamine green (Wolffsohn et al., 2017). A slit lamp is used for many of these tests, and it can be used for evaluating other ocular tissues (cornea, iris, lens, retina). However, the availability of diagnostic and treatment tools will be limited in long-haul spaceflights due to payload (volume and mass) and opportunities to replace diagnostic products or treatments are scarce.

The current diagnostic equipment on the ISS includes an ophthalmoscope with downlink video function as well as a blue light filter and fluorescein strips. A slit lamp is not available, but astronauts are trained to use an ophthalmoscope to perform a complete primary ocular examination with lid eversion. Fluid shift may confound the diagnosis due to congestion of conjunctival blood vessels, causing “red eye” (Marshburn et al., 2019). Installing more advanced ocular surface diagnosis technology on the ISS or on future spaceships and stations would present a challenge. Automated NIBUT (non-invasive tear breakup time), tear film interferometry and osmolarity test devices are too bulky or use disposables and are therefore unsuitable for being part of the payload. Rather, small, multipurpose systems and self-administered testing should be considered. Infrared imaging, for example, has shown potential as a non-invasive and disposable-free diagnostic tool and has already been used on the ISS for other purposes (Howell et al., 2008; Shah and Galor, 2021). Screen and smartphone-based tests for blinking and evaluating the eye are developing rapidly, and some of these could be considered as they become available (Wolffsohn et al., 2018; Okumura et al., 2022).

## 5 Treatment of tear film problems in space

**FIGURE 1:**
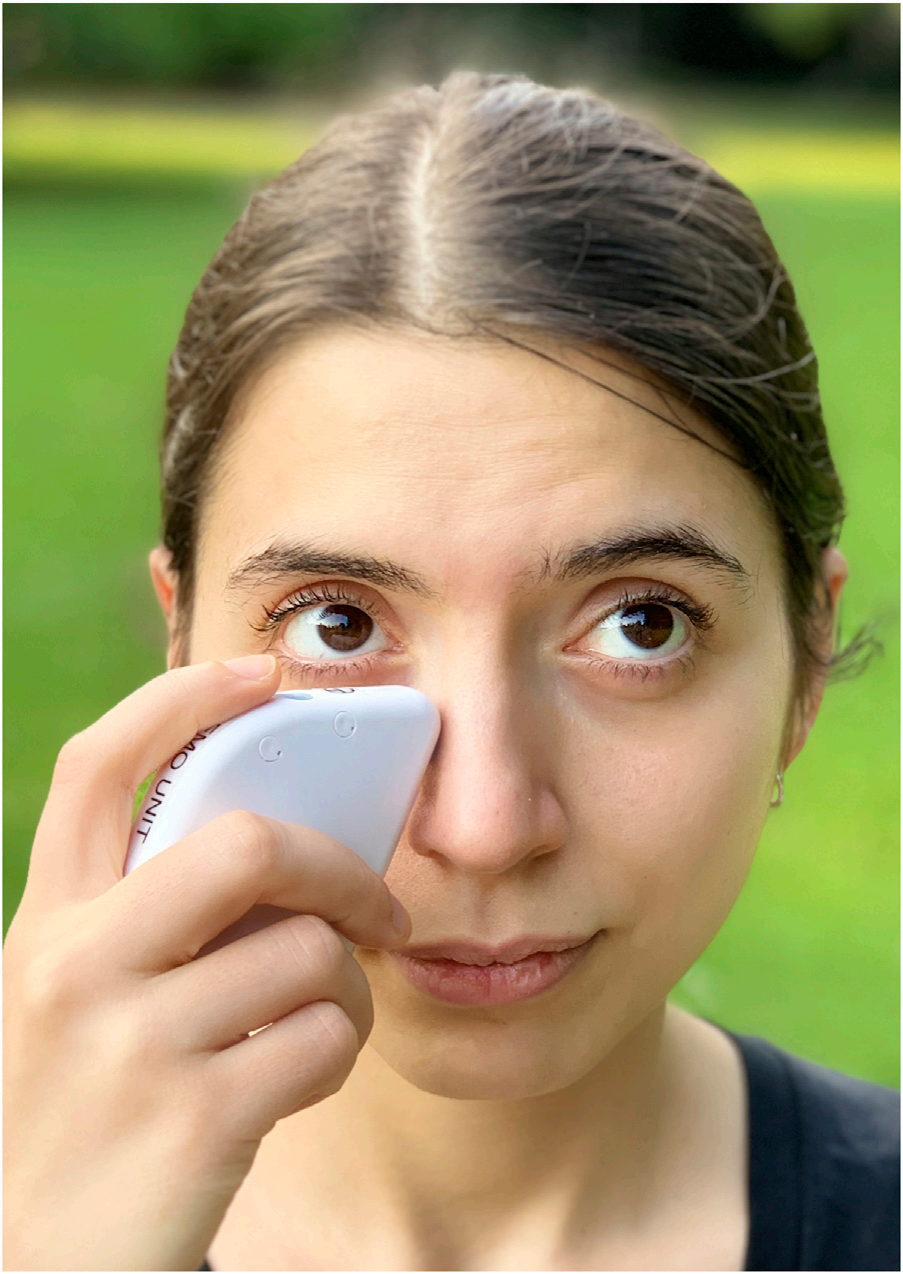
Application of iTear100 neurostimulation system.

Eyedrops are the most common form of treatment for tear problems on Earth (Jones et al., 2017). These will be inappropriate for long-haul spaceflights because of payload and use-by dates. The application of eyedrops in microgravity would also be a major issue because “dropping” them into the eye is challenging. The fluid must be “wicked” into the eye using surface tension forces by making direct contact with the dropper bottle, thus contaminating the bottle in the process. Ointments and gels are easier to apply and are currently in use on the ISS for eye infections but would impair vision when used for lubrication. Therefore, other methods related to restoring the physiology of the tear film should be used. One possible option is ocular neurostimulation. While early devices used electricity and disposables (TrueTear, Allergan Inc.), current technology (iTear100, Olympic Ophthalmics Inc.; Figure 1) externally stimulates the nasociliary nerve through vibration to immediately induce lacrimation and potentially Meibomian gland secretion (Farhangi et al., 2019; Ji et al., 2020). This means that one device could be used by the entire crew. Other “low tech” treatments which have proven effective on Earth are blink exercises, heating and massage of Meibomian glands, and cleaning the area around the eyes (Bitton et al., 2019; McMonnies, 2021). However, unlike neurostimulation these need to be repeated on a regular basis to start providing relief.

## 6 Environmental hazards associated with space travel

The interior of spacecraft and space stations are likely to have an impact on the tear film. This is because of strong air currents (air exchanged 45–77 times per hour in crew quarters) coupled with a relative humidity of about 60% (ISS), increased CO_2_ exposure, artificial lighting, cosmic radiation, and extensive screen work (Gamus et al., 1994; Scull et al., 1998; Uchino et al., 2013; Stenger et al., 2017; Georgescu et al., 2021) which increase tear film evaporation and inflammation of the ocular surface. These factors need to be addressed to minimize the risk of ocular surface damage.

Astronauts are either inside a spaceship or space station, or in special extravehicular activity (EVA) suits with no opportunity to move to a different environment. That way, they are constantly exposed to aerosols and bioaerosols (airborne particles containing microbes such as bacteria, fungi, and viruses) which accumulate in the cabin (Harrad et al., 2023). Larger particles which would settle out on Earth also harbor higher concentrations of microbes (Haines et al., 2019). Furthermore, there is research pointing towards alterations of virulence of pathogens in microgravity (Velez Justiniano et al., 2023).

On the ISS, high-efficiency particle air filters remove particles with a minimum efficiency of 99.97%, which diminishes the chance of these particles entering astronauts’ eyes. The ISS also has eye-wash devices available to flush debris from the eye (James and Ryder, 2019).

On Earth, extensive use of visual display terminals (VDT), such as computer screens, is a major cause of DED. VDT use adversely affects blinking and Meibomian glands, NIBUT, ocular comfort and causes signs of ocular surface damage (Fjaervoll et al., 2022). Breaks from using screens and blink exercises could provide relief (Wolffsohn et al., 2023).

Sleep is needed for the ocular surface to regenerate (Li et al., 2022). Lack of sleep or poor sleep quality are risk factors of DED (Ayaki et al., 2016; Galor et al., 2023). Astronauts often report difficulty falling asleep and staying asleep, and disruptions to their circadian rhythms. This is mainly due to the lack of a natural day-night cycle, as well as the noise and lighting conditions (especially blue light). To mitigate these issues, astronauts typically follow a carefully designed sleep schedule and may use sleep aids such as eye masks and earplugs (Allen et al., 2016; Barger et al., 2020). Recently, dynamic solid-state lighting was installed on the ISS as a countermeasure for circadian misalignment and sleep disruption (Brainard et al., 2016; Rahman et al., 2022).

In addition to sleep disorders, behavioral and mood disorders occur with increasing mission length (Clement et al., 2020). They are associated with dry eye and *vice versa*, though the exact mechanism is yet to be determined. One possible mechanism is that symptoms of DED affect the daily lives of patients, thus affecting their mood (An and Kim, 2022; Tsai et al., 2022; Galor et al., 2023). The management of mood disorders in space is complex and ranges from astronaut selection as a preventative measure to in-flight private videoconferences with behavioral health specialists (Sipes et al., 2021).

## 7 Discussion

Current evidence suggests that DED will be an issue during long-haul spaceflights, and it will need to be addressed to prevent potential consequences for astronaut health and performance. Experience from ISS crew indicates that ∼30% of astronauts are affected (Reschke et al., 2016), and this warrants systematic investigation that includes the type of DED afflicting astronauts, identification of factors contributing to it, and modes of treatment suitable for space travel. Blinking behavior and tear film dynamics could be studied on a parabolic flight to uncover immediate effects of microgravity. Long-term effects, on the other hand, could be investigated by performing tear film sampling on astronauts in addition to blink and tear film tests.

With NASA planning a return to the Moon as soon as 2024, the potential dangers of the lunar environment need to be accounted for (Smith et al., 2020). Extraterrestrial dust is a potential threat, e.g., acute exposure to lunar dust causes ocular irritation (Meyers et al., 2012) but is not directly toxic. Lunar dust tends to be especially jagged due to the absence of weathering (James and Kahn-Mayberry, 2011; Pohlen et al., 2022; Miranda et al., 2023). In the Apollo missions, numerous astronauts reported ocular erythematous and watery eyes with decreased vision after exposure to lunar dust following its introduction to the lunar module upon return from EVAs (Scheuring et al., 2008). Martian dust is potentially a bigger threat to ocular health because it contains reactive perchlorates (Davila et al., 2013; Crotts, 2014).

Goggles could be worn by astronauts when acute exposure is expected. Once exposed, while eye irrigation would help, repeated irrigation, e.g., after every EVA, would most likely remove the protective mucin cover of the ocular surface increasing the risk of DED and infection (Naderi et al., 2020). However, 1 month of daily eye irrigation with a commercial eye wash solution containing vitamins, chondroitin sulfate and other supplements did not show detrimental effects (Yazu et al., 2019). It is yet to be determined if repeated flushing with recycled water as used in the ISS would have any detrimental effects on eye health (Straub et al., 2013). Alternatively, neurostimulation (see above) to promote tearing and natural flushing would be a possibility worth investigating.

In conclusion, there are still significant knowledge gaps when it comes to ocular surface health in space. These should be addressed by future research.
